# Bioprinting of Matrigel Scaffolds for Cancer Research

**DOI:** 10.3390/polym13122026

**Published:** 2021-06-21

**Authors:** Paola De Stefano, Francesco Briatico-Vangosa, Elena Bianchi, Alessandro Filippo Pellegata, Ariel Hartung de Hartungen, Pietro Corti, Gabriele Dubini

**Affiliations:** 1 Laboratory of Biological Structure Mechanics (LaBS), Department of Chemistry, Materials and Chemical Engineering ‘Giulio Natta’, Politecnico di Milano, Piazza Leonardo da Vinci 32, 20133 Milan, Italy; elena1.bianchi@polimi.it (E.B.); alessandro.pellegata@polimi.it (A.F.P.); ariel.hartungdehartungen@mail.polimi.it (A.H.d.H.); pietro2.corti@mail.polimi.it (P.C.); gabriele.dubini@polimi.it (G.D.); 2 Polymer Engineering Group, Department of Chemistry, Materials and Chemical Engineering ‘Giulio Natta’, Politecnico di Milano, Piazza Leonardo da Vinci 32, 20133 Milan, Italy; francesco.briatico@polimi.it

**Keywords:** 3D bioprinting, Matrigel^®^, volumetric dispensing

## Abstract

Cancer is one of the most life-threatening diseases worldwide. Despite the huge efforts, the failure rate of therapies remains high due to cells heterogeneity, so physiologically relevant models are strictly necessary. Bioprinting is a technology able to form highly complex 3D tissue models and enables the creation of large-scale constructs. In cancer research, Matrigel^®^ is the most widely used matrix, but it is hardly bioprinted pure, without the use of any other bioink as reinforcement. Its complex rheological behavior makes the control with a standard bioprinting process nearly impossible. In this work, we present a customized bioprinting strategy to produce pure Matrigel^®^ scaffolds with good shape fidelity. To this aim, we realized a custom-made volumetric dispensing system and performed printability evaluations. To determine optimal printing parameters, we analyzed fibers spreading ratio on simple serpentines. After identifying an optimal flow rate of 86.68 ± 5.77 µL/min and a printing speed of 10 mm/min, we moved forward to evaluate printing accuracy, structural integrity and other key parameters on single and multi-layer grids. Results demonstrated that Matrigel^®^ was able to maintain its structure in both simple and complex designs, as well as in single and multilayer structures, even if it does not possess high mechanical strength. In conclusion, the use of volumetric dispensing allowed printing pure Matrigel^®^ constructs with a certain degree of shape fidelity on both single and multiple layers.

## 1. Introduction

Cancer is one of the most life-threatening disease worldwide, accounting for an estimated 9.6 million deaths in 2018 [1]. Despite huge efforts, the failure rate of therapies remains still high due to cells heterogeneity, which leads to the development of drug-resistance mechanism [2,3]. Therefore, more complex and physiologically relevant models are strictly necessary to obtain a deeper understanding of cancer evolution.

Moreover, 3D models, contrarily to 2D monolayer cell cultures, provide more accurate representations of cancer tissues in terms of tumor microenvironment and biological behavior by mimicking 3D complexity, which is crucial for developing early diagnosis and treatment strategies for cancer [2]. Among all of them, organoids possess excellent potential for studying development and disease mechanism. However, current 3D in vitro models suffer from a number of significant limitations, such as oversimplified structures, limited vascularization [4], and limited dimensions of tissue constructs [5].

To overcome these problems, bioprinting can be considered a potential solution, as it is able to form highly controllable cancer tissue models thanks to the precise control of cell distribution within 3D space. Moreover, it enables the creation of large-scale constructs using either single-cells or organoids [2,4,5].

In cancer research, Matrigel^®^, a natural hydrogel ECM purified from Engelbreth-Holm-Swarm mouse sarcoma, is the most widely used matrix for culture of 3D organoids [5,6], thanks to its biologically active nature. These cellular constructs, whose dimensions are about hundreds of microns, usually grow inside small droplets generated using standard pipetting systems and represent a good solution for short-term cultures. However, to study drug-resistance mechanism and tumor evolution, biologists require novel 3D large-scale cancer models. These structures would be preferably realized with Matrigel^®^, but its complex rheological behavior and low mechanical properties impede to bioprint it pure. For these reasons, it is often combined with other bioinks [5,7,8,9] as support materials, even if they introduce additional biological variabilities, which make them a suboptimal choice in the field of 3D culture of organoids.

Indeed, it is not possible to extrude Matrigel^®^ with a standard pneumatic-driven dispensing system as it is ejected out of the syringe with an uncontrollable behavior as soon as pressure is applied. This phenomenon seems to be similar to the “spurt” effect reported by polymeric melts, in which above a certain critical pressure value, flow rate abruptly increases [10,11,12]. Instead, volumetric control allows uncoupling the extrusion of Matrigel^®^ from the pressure generated in the dispenser, which directly depends on the rheological properties of the matrix, working conditions and nozzle geometry.

To the best of our knowledge, only J. E. Snyder et al. [13] successfully bioprinted pure Matrigel^®^. They bioprinted Matrigel^®^ containing liver cells into a PDMS microfluidic chip, by using a temperature-controlled cell printing system that prevented gelation prior to Matrigel^®^ extrusion. In this way, the shape of the structures was given by the confinement of the extruded matrix into a pre-defined cavity. Consequently, cell cultures could only be performed inside the chip. Instead, 3D bioprinting of free-standing Matrigel^®^ constructs remained an open point.

In our work, we address it by presenting an alternative bioprinting strategy to produce Matrigel^®^ scaffolds. This method, contrarily to what is reported in the literature, allows obtaining free-standing structures with good shape fidelity.

## 2. Materials and Methods

### 2.1. Bioprinting System

**Figure 1:**
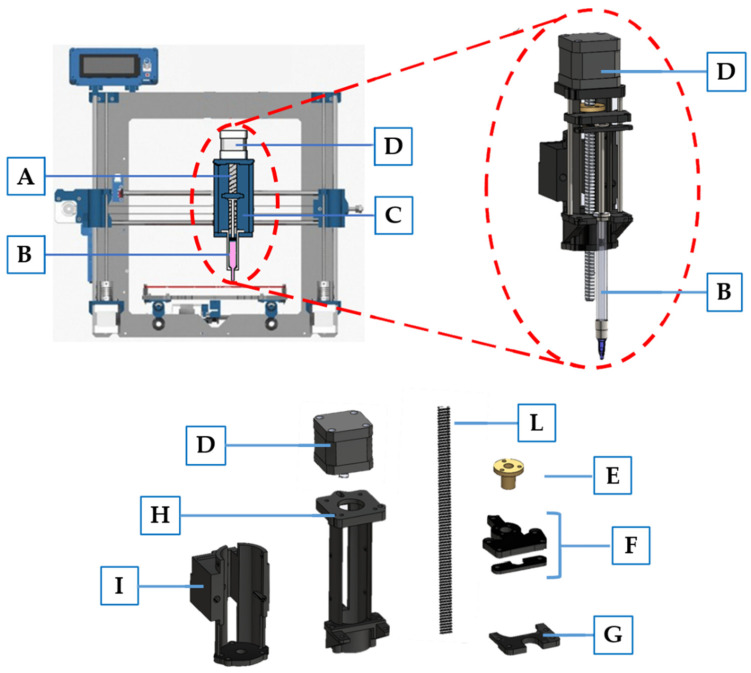
Schematic representation of modified 3D printer Prusa I3. The main components include: (**A**) linear actuator designed to push syringe plunger; (**B**) syringe containing Matrigel; (**C**) syringe housing; (**D**) stepper motor; (**E**) ball screw; (**F**) syringe plunger holder connected to the ball screw to achieve movement; (**G**) syringe holder; (**H**) linear actuator structure; (**I**) adapter to connect the linear actuator to the 3D printer moving part; (**L**) M8 threaded rod with a 2 mm pitch.

Bioprinting was performed using a commercial 3D Fused Deposition Modeling (FDM) printer Prusa i3 (Prusa Research, Prague, Czech Republic), specially modified to achieve a volumetric dispensing system. In particular, the printhead, initially designed to heat and extrude a plastic filament, was modified with a custom-made syringe pumping system, comprised of a linear actuator connected to a syringe housing (Figure 1), designed with Solidworks 2020 and 3D printed in PLA. A stepper motor (17HS16-2004S, Quimat, Hong Kong, China) was attached to the syringe adapter in order to push the plunger of a syringe containing the bioink. To achieve a counterclockwise rotation—and therefore the proper movement of the linear actuator—the four wires were reversed. Furthermore, to obtain a functioning system, we also disabled the extruder and printing plate heating system, as they were no more required. In Table 1, we report the starting and the ending gcode that we used to bioprint our constructs.

### 2.2. Bioink Preparation

For these experiments, ThermoFisher Scientific Matrigel^®^ (LOT 0202003, Corning, NY, USA) was used. It was maintained at 4 °C to ensure liquid-state and proper loading inside a 1 mL glass syringe (Gas Tight, Hamilton Company, Reno, NV, USA) minimizing the presence of air bubbles. Then, it was placed into an incubator at 37 °C for 15 min to obtain complete Matrigel^®^ gelation. Finally, the syringe was mounted on Prusa i3 adapter for bioprinting.

### 2.3. Optimization of the Printing Process

For fiber bioprinting optimization, we performed two major steps: definition of optimal flow rate and printing speed. Firstly, we printed serpentines with three different flow rates (43.34 ± 5.77, 86.68 ± 5.77 and 116.69 ± 5.77 µL/min) corresponding to E100, E150 and E200 in the gcode file. After defining optimal flow rate, four printing speeds (5, 10, 15 and 20 mm/s) were evaluated. All tests were performed at room temperature using a 25G (inner diameter 0.26 mm) plastic conical needle and glass microscope slides as printing surface. CAD files were designed in Solidworks 2020 (Dassault Systèmes SOLIDWORKS Corp., Waltham, MA, USA) and processed with Ultimaker Cura 4.7. Images of the fibers were acquired with an inverted microscope (Olympus IX73 SC180 CSD) at 4× magnification and analyzed with ImageJ (NIH, Bethesda, MD, USA) software [14].

For each serpentine printed, we analyzed their ability to maintain the desired shape upon deposition by computing the spreading ratio Equation (1) [15]:

(1) $$\mathrm{Spreading}\;\mathrm{ratio}=\frac{\mathrm{Filament}\;\mathrm{Diameter}\;}{\mathrm{Needle}\;\mathrm{Diameter}}$$

**Figure 2:**
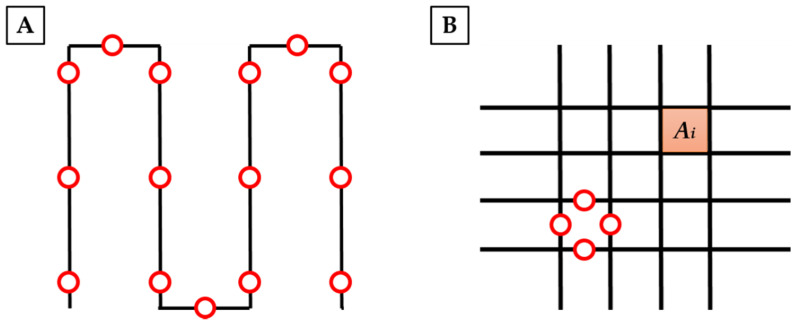
Schematic representation of fiber diameter and pores evaluation. In panel (**A**) red circles indicate points in which fibers were measured. In panel (**B**) an orange square shows an example of a printed area taken into consideration for printing accuracy evaluation, while red circles indicates the main points used to estimate fiber diameters in grid structures. Those analyses were performed for each pore.

Fifteen measures of the fibers were taken (Figure 2A) and then, the average spreading ratio was evaluated. According to the results, we defined optimal parameters in order to achieve continuous, homogeneous fiber deposition, and the lowest spreading ratio.

### 2.4. Printing Accuracy

To evaluate the printability of complex structures, we performed a preliminary analysis on single-layered squared grids with varied pores side ranging from 4 to 6 mm, and layer height of 0.4 mm. For printing, we used optimal parameters defined as described in paragraph 2.3. Images were obtained using an inverted Microscope (Olympus IX73 SC180 CSD, Olympus Corp., Tokyo, Japan) with a 4× magnification and analyzed using ImageJ software. For each print, we performed morphological characterization of the pores in order to assess the printing accuracy and shape fidelity. Specifically, we calculated printing accuracy using the following Equation (2) [16]:

(2) $$\mathrm{Printing}\;\mathrm{Accuracy}\;\left[\%\right]=\left[1-\;\frac{\left|A_{i}-A\right|}{A}\right]\;\times \;100$$

For each grid, the printed area A_i_ of each pore was compared to the designed area A (Figure 2B). The average printing accuracy was then estimated.

Instead, as regards printability index (Pr), we used Equation (3) [17]:

(3) $$\mathrm{Pr}=\frac{L^{2}}{16A}$$

where L is the average perimeter of the pore, while A is the average area of the pore. Pores with perfect squared shape have a Pr index equal to 1. Instead, circular pores have Pr < 1, while irregular shapes have Pr > 1. For each grid, each pore was considered in the calculation. Then, the average printability index was determined.

In order to assess the capability of the bioink to retain their structure when printed over multiple layers, we printed six layer grids with two different shapes: square and circle. In this case, we considered three main parameters in the evaluation: layer height, shape fidelity [18] and structural integrity [19]. In particular, for the last two indexes, we used the following Equations (4) and (5):

(4) $$\mathrm{Shape}\;\mathrm{fidelity}\;\left[\%\right]=\frac{\mathrm{Printed}\;\mathrm{pore}\;\mathrm{area}}{\mathrm{Designed}\;\mathrm{pore}\;\mathrm{area}}\;\times \;100$$

(5) $$\mathrm{Structural}\;\mathrm{integrity}\;[\%]=\frac{\mathrm{Measured}\;\mathrm{height}}{\mathrm{Desired}\;\mathrm{height}}\;\times \;100$$

For both single and multi-layer structures, we also evaluated the mean fiber diameter of each pore (Figure 2B).

### 2.5. Rheological Characterization

The rheological behavior of the bioink was characterized using an Anton Paar MCR 502 rheometer (Anton Paar, Graz, Austria) with parallel-plate configuration (25 mm diameter, 0.45 mm gap). Temperature was controlled during the entire test using a Peltier plate and hood. In order to obtain proper loading of the sample, a Matrigel^®^ droplet of 600 µL was pipetted on a Parafilm sheet four hours before the experiment and stored in a freezer at −4 °C. At the beginning of the test, the sample was gently positioned between the pre-cooled rheometer plates, at 2 °C, using a spatula. A wet paper was then placed inside the test hood far from the sample, to prevent dehydration. In order to investigate the effect of temperature on the rheological behavior of the bioink, the same thermal history as during the bioprinting experiment was applied to the sample. An initial oscillatory test (1% strain, 1 rad/s angular frequency) was carried out while heating the sample up to 37 °C. After this temperature was maintained for 15 min, a frequency sweep test was conducted from 0.1 to 10 rad/s (1% strain). Then, the sample was cooled down to 20 °C and a second frequency sweep test was performed at this temperature (strain of 1%, 0.1–10 rad/s). Finally, steady state shear-viscosity tests were conducted at 20 °C in the 0.1–500 s^−1^ shear rates range, first scanning it from the lower to the higher value and then going back from the higher to the lower. In this test, viscosity was measured at logarithmically spaced shear rate points.

### 2.6. Cell Viability

Murine prostate cancer cells (Pten-/-P53-/-) were expanded in monolayer cultures, containing complete Dulbecco modified Eagle’s medium (DMEM, Gibco^TM^, ThermoFisher Scientific, Waltham, MA, USA) culture medium, supplemented with 10% FBS, Penicillin-Streptomycin and L-Glutammine. They were maintained at 37 °C and 5% CO_2_. At 90% confluence, they were washed with PBS (MicroGem, TL1006-500ML, VWR, Radnor, OH, USA), trypsinized and resuspended in Matrigel^®^ solution at a cell density of 10^6^ cells/mL. Then, it was loaded into a 1 mL glass syringe (Gas Tight, Hamilton Company, Reno, NV, USA) minimizing the presence of air bubbles. To avoid Matrigel^®^ gelation, all materials were kept at 4 °C. At the end of the preparation, the syringe was placed into an incubator at 37 °C for 15 min to obtain complete Matrigel^®^ gelation. Finally, it was mounted on Prusa i3 adapter for bioprinting and three circular six-layer grids (1.5 cm diameter) were printed into a 6 well plate, using a flow rate of 86.68 µL/min, a printing speed of 10 mm/s and a 25G conical plastic needle. Three drops of 100 µL were manually deposited into a separate 6-well plates as controls. Afterwards, 2 mL of DMEM were added to each well and plates were placed into the incubator at 37 °C and 5% CO_2_.

Cells images were acquired with an inverted microscope (Olympus IX73 SC180 CSD, Olympus Corp., Tokyo, Japan) at 10× and 20× magnification 4 h after the extrusion process. To evaluate cell viability, alive and dead cells were counted. Each construct was mechanically disrupted by pipetting 2 mL of ice-cold PBS solution in each well and then, centrifuged for 5 min. After, 10 µL of the final suspension were mixed with an equal amount of trypan blue for live-dead cell counting by using the Bürker counting chamber.

### 2.7. Statistical Analysis

Data were expressed as mean ± standard deviation. Statistical analyses were performed using GraphPad Prism 7.00 and differences were evaluated by a non-parametric *t*-test. A value of *p* < 0.05 was considered as statistically significant.

## 3. Results and Discussion

Matrigel^®^ is an ECM mixture primarily constituted by collagen type IV, laminin, entactin and perlecan. Thanks to its composition, it resembles complex basement membrane environments that could be found in many normal tissues [20]. Moreover, even if its exact composition is not well defined as it is derived from mouse tumor cells, researchers continue to use it for cell cultures owing to its availability, ease of use and versatility for culturing different types of cells [21]. Moreover, it is able to facilitate cells self-organization into structures that closely mimic the features of in vivo tissue [6], which makes it the most widely used matrix for 3D organoids cultures. It is also used in 3D bioprinting applications. However, it is commonly used in combination with other bioinks, because it possesses a complex rheological behavior that makes it hardly bioprintable with common bioprinting strategies. It is used to improve biocompatibility, as many hydrogels lack essential protein components, compromising performances. Therefore, we proposed a modified strategy to bioprint pure Matrigel^®^, analyzing the impact of volumetric dispensing. In particular, in order to obtain 3D structures, we first assessed printability and shape fidelity, as key aspects in the development of a bioink.

### 3.1. Optimization of the Printing Process

The first step was to define the optimal printing parameters in order to obtain a continuous and homogenous filament with good shape fidelity. To reach this main goal, serpentines were printed with different feeding rates and the width of the printed fibers were measured using ImageJ. 

**Figure 3:**
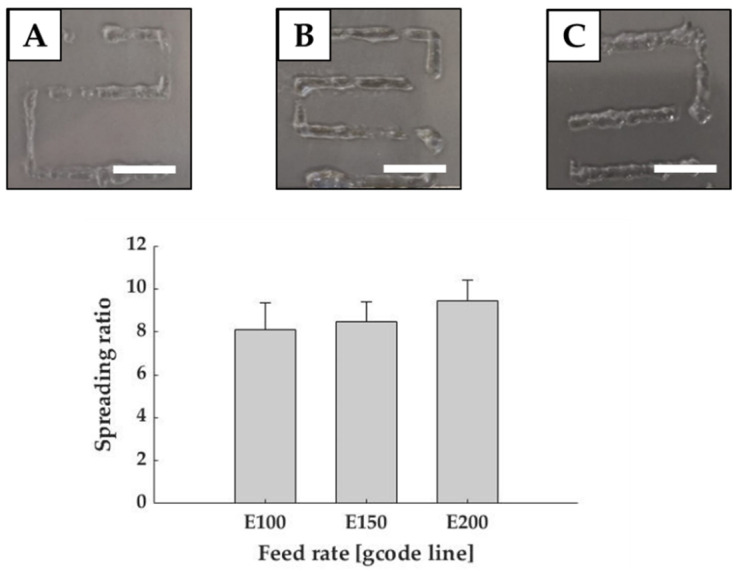
Printability evaluation in terms of feed rate. Graph shows the spreading ratio of Matrigel^®^ fibers. Representative images are reported for E100 (**A**), E150 (**B**), E200 (**C**). Scale bar: 1 cm.

Figure 3 shows that the spreading ratio progressively increased accordingly to the feed rate, but fibers became more homogeneous even if the filaments presented some discontinuities. In selecting the best parameter, the lowest spreading ratio (Equation (1)) is desirable as it allows creating cell laden hydrogel structures with high precision. However, standard deviation must be considered because fibers must be also homogenous. The lowest spreading ratio was reported with a flow rate of 43.34 ± 5.77 µL/min, corresponding to gcode E100, but it possessed the highest standard deviation. Instead, fibers achieved with flow rates of 86.68 ± 5.77 µL/min (E150) and 116.69 ± 5.77 µL/min (E200) were more homogeneous with standard deviation equal to 1.89. Despite the variations, all values remained comparable with those reported in the literature for common bioinks, such as Low MW Alginate crosslinked with Calcium Chloride [22]. Considering all these aspects, fibers obtained with 86.68 ± 5.77 µL/min (E150), which had a spreading ratio of 8.48 ± 1.82 (mean fiber diameter of 2.21 ± 0.47 mm), were considered as the best results in terms of both spreading ratio and homogeneity. This feed rate was used for the following analyses.

**Figure 4:**
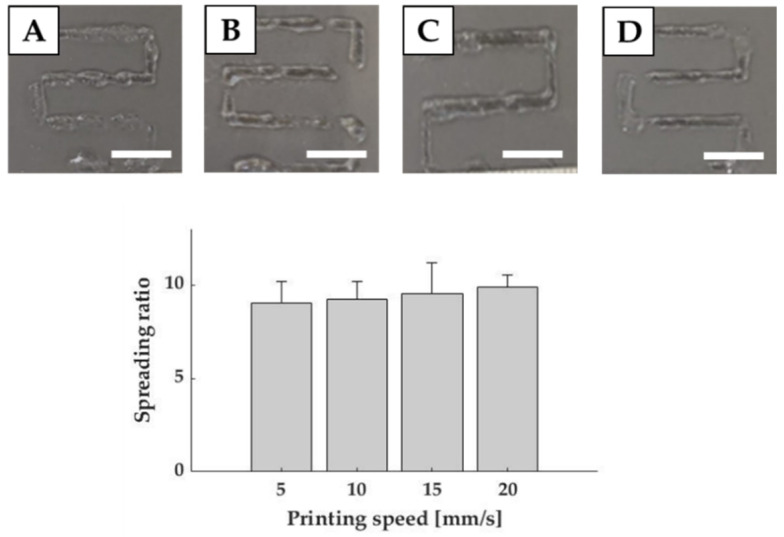
Printability evaluation in terms of printing speed. Graph shows the spreading ratio of Matrigel fibers. Representative images are reported for 5 mm/s (**A**), 10 mm/s (**B**), 15 mm/s (**C**) and 20 mm/s (**D**) speeds. Scale bar: 1 cm.

To establish the optimal printing speed, we investigated four different velocities ranging from 5 to 20 mm/s, using the selected feed rate. In this case, results reported only slight variations among the different values. Indeed, the lowest spreading ratio, obtained with 5 mm/s, was 7.54 ± 1.66 (mean fiber diameter of 1.96 ± 0.43 mm), which differed from the values obtained for 10 mm/s and 20 mm/s only for 2.5% and 8.6% respectively (Figure 4). However, as stated for feed rate evaluation, lower standard deviations were recorded for 10 and 20 mm/s. Therefore, in order to determine the optimal printing speed, we selected the lowest spreading ratio with the lowest standard deviation (9.24 ± 1.84), corresponding to a mean fiber diameter of 2.40 ± 0.48 mm, which was achieved with 10 mm/s.

### 3.2. Printing Accuracy on Single Layer Constructs

In order to evaluate the capability of Matrigel^®^ to be used for complex structures, we evaluated printing accuracy (Equation (2)) in terms of difference between the printed and theoretical area. By estimating this index, we evaluated the capability of the system to maintain an open area compared to the designed one. For this purpose, we analyzed single layer squared grids with side square pore ranging from 4 to 6 mm. 

D shows that larger pores corresponded to better printing accuracy. Indeed, square pores of 6 mm side accounted for 78.19% ± 18.41%, while square pores of 4 mm side reached 34.30% ± 15.99%, showing less precision and shape fidelity with respect to the CAD model.

**Figure 5:**
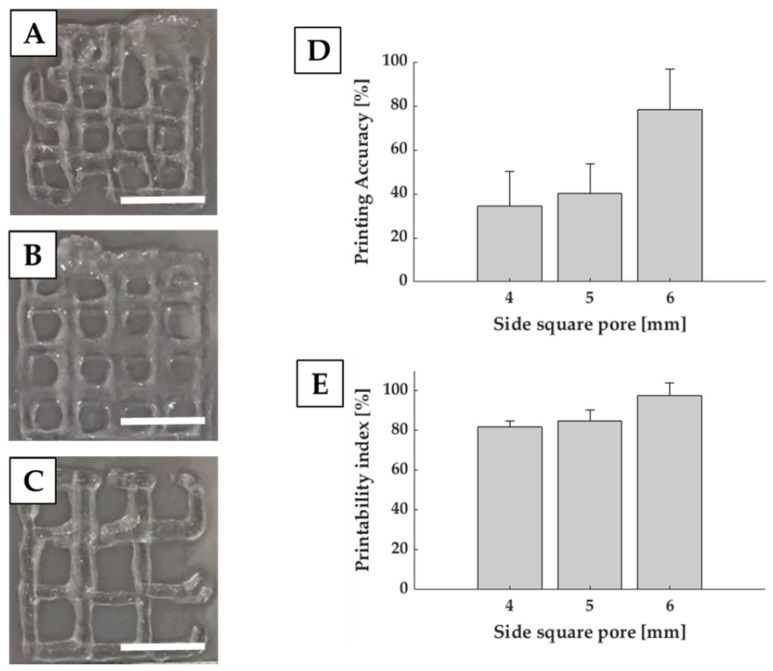
Printability evaluation of single layer grids. Representative images are reported for square pores of 4 (**A**), 5 (**B**) and 6 (**C**) mm side. Graphs show the printing accuracy (**D**) and printability index (**E**). Scale bar: 1 cm.

Together with printing accuracy, we also evaluated printability index (Pr) (Equation (3)), which is related to the capability of the structure to retain a squared pore. Results reported in Figure 5E shows, for square pores of 6 mm side, a Pr close to 1, indicating that the squareness of the pore is preserved even if smaller than the designed one. Reduction in the pore dimension induces a Pr decrease. However, it still remains over 0.8, which indicates a good capability of the constructs to retain their structures compared to CAD models. Printability index is a semiquantitative evaluation, based on the circularity of the printed fibers and shape fidelity of the pore. It gives an indication of the ink stabilization after dispensing and can provide an estimation of the shape fidelity in the x–y plane [23].

### 3.3. Printing Accuracy on Multi-Layer Scaffolds

**Figure 6:**
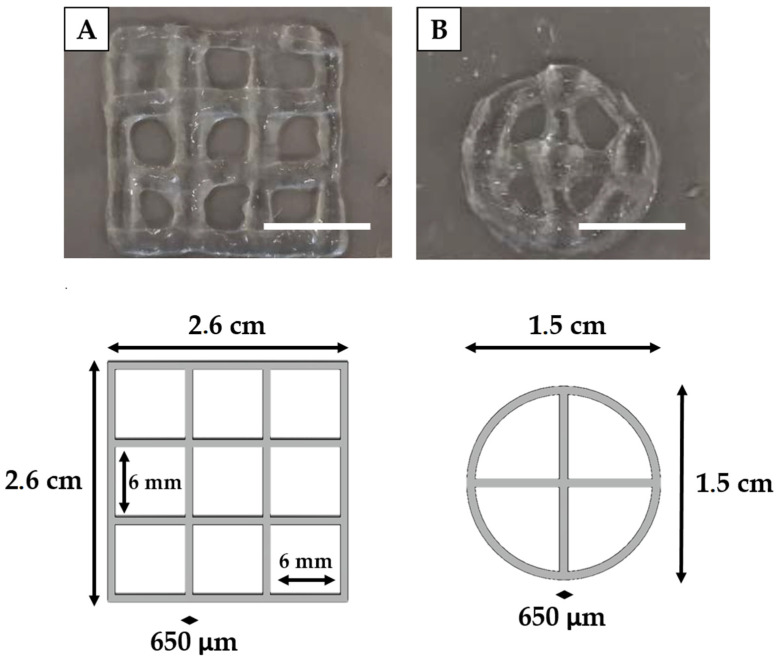
Printability evaluation of multi-layer grids. Representative images, reported for square (**A**) and circular (**B**) grids, are compared with the respective CAD models. Scale bar: 1 cm.

Once achieved the proper deposition of single layer structures, it is important to evaluate printability on multi-layer constructs. In this case three key parameters must be evaluated: the geometric accuracy, in terms of shape fidelity (Equation (4)), layer stacking and structural integrity (Equation (5)) [23]. By comparing the printed structures with CAD models (Figure 6) and with the results achieved with single layer constructs, we were able to determine the capability of the fibers to maintain their shape in case of multi-layer printing. Indeed, the mean fiber diameter obtained for squared and circular structures (2.71 ± 0.41 mm and 3.4 ± 0.74 mm) was comparable with the one obtained in the previous analyses, demonstrating consistency in the bioprinting process also in the case of multi-layer structures and with different geometries.

Moreover, shape fidelity was about 30% for both structures. This percentage was also associated with a high standard deviation, which might be due to undesired movements of the custom-made extruder. This possible layer misalignment during bioink deposition may be solved by further stabilizing the extruder. This phenomenon could be an explanation of the rapid decrease in shape fidelity for squared grids printed on six layers compared to the single layer one. Hence, filaments tended to collapse and to merge with each other. However, filaments were still capable of maintaining the pore shape, although becoming smaller and rounder. As regards, instead, structural integrity, it reached at least 50% (Table 2), which was in accordance with the expectations, considering Matrigel low mechanical properties. However, results were higher than those reported in literature, in which the structure totally collapsed. In the literature, for example, the three layer Matrigel^®^ grid realized by Schmidt et al. [24] was unable to retain the desired shape, flattened onto the substate, and occluded the pores of the grid.

Furthermore, our bioprinted structures have also dimensions comparable with those reported in the literature [13,15], so we are confident that they would be suitable to produce long-term large scale 3D in vitro cancer models.

### 3.4. Rheological Characterization

**Figure 7:**
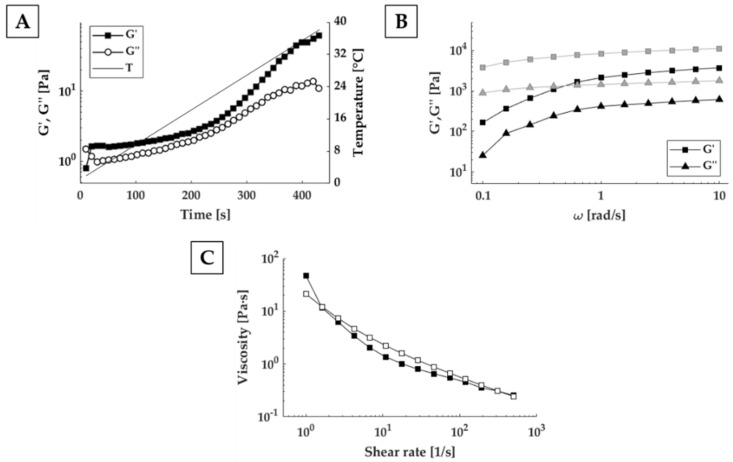
Matrigel^®^ rheological characterization during preparation and extrusion phase. (**A**) Evaluation of G’ and G” value over time at 1 rad/s for thermal history investigation. (**B**) Frequency sweep test at 20 °C (grey) and 37 °C (black). (**C**) Steady state viscosity measurements ranging from 0.1 to 500 s^−1^ (filled symbols) and from 500 to 0.1 s^−1^ (open symbols).

To obtain optimal use of Matrigel^®^ in bioprinting applications, understanding and characterizing rheological properties is necessary. Considering the effect of temperature on Matrigel^®^ crosslinking, the first step was the investigation of the effects of the thermal history the material undergoes before bioprinting. By increasing temperature from 2 to 37 °C at 5 °C/min, we identified the gelation point, as point of crossover between the conservative (G’) and dissipative (G”) components of the complex shear modulus around 6 °C, which is in line with the work of Kane et al. [25]. Below this value, Matrigel^®^ is in liquid-state, while above it, it starts exhibiting a solid-like behaviour. However, as reported in Figure 7A, the difference between storage (G’) and loss (G”) modulus remained small, making it difficult to manipulate the material without an accurate temperature control system. At temperature higher than around 17 °C, instead, the two curves started to diverge suggesting the formation of a consistent gel. Since Matrigel was gelled in the incubator at 37 °C, and then extruded at room temperature, we compared mechanical properties at both temperatures. Looking at the Figure 7B, the ratio between G’ and G” at 20 and 37 °C is approximately equal. However, mechanical properties are higher at 20 °C than 37 °C. With the initial gel properties defined, to assess bioink printability, we needed to understand the response of the material during the extrusion stage. For this purpose, we analyzed viscosity dependency from the shear rate by recording the flow curves (Figure 7C). Increasing the shear rate from 0.1 to 500 s^−1^, viscosity rapidly dropped from 46.6 to 0.25 Pa·s. The material viscosity trend was similar on both increasing and decreasing shear rates, suggesting that no permanent structural variations occur during shearing.

### 3.5. Cell Viability

**Figure 8:**
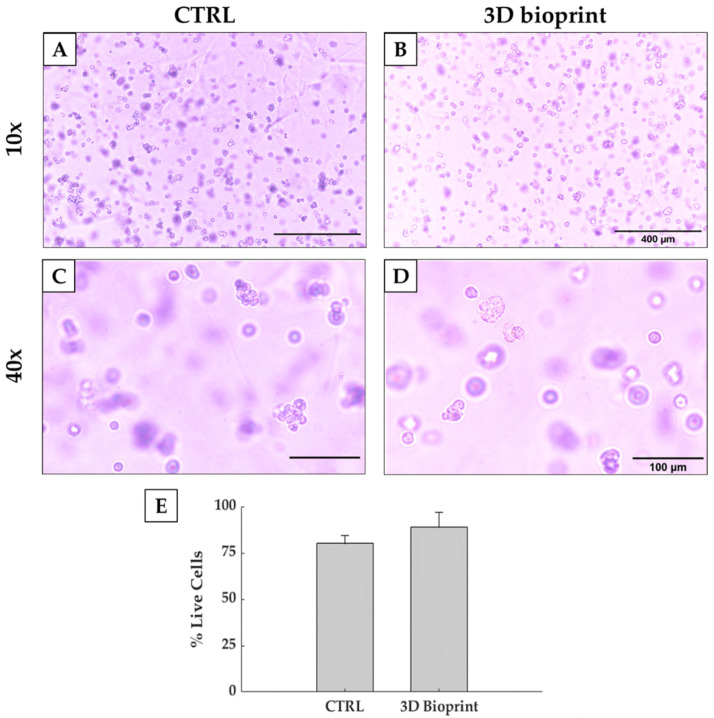
Optical images taken from a control (**A**,**C**) and a bioprinted Matrigel^®^ construct (**B**,**D**). Pictures in the upper part of the scheme are acquired at 10× magnification (scale bar: 400 µm), while on the bottom part some enlargement of the images acquired with 20× magnification is reported (scale bar: 100 µm). (**E**) Percentage of live cells of the cells in bioprinted and non-bioprinted constructs.

Shear stress is a key parameter in cell biology, as it influences both cell viability, but also cell signaling and protein expression. Indeed, excessive shear stresses can disrupt cells membrane and induce cell death. Shear stress is strictly related to multiple parameters, such as flow rate, nozzle shape and diameter and bioink viscosity. Common bioprinting methods use air pressure to extrude a filament, but this method is unable to accurately control shear inside the cartridge. The same problem is also present with manual pipetting, where biologists do not have control of shear stresses applied to cells. On the contrary, the use of a volumetric dispensing system allows a better control of the extrusion process and the shear stress levels. Indeed, in our test we compared manual deposition, considered as control, with the volumetric-controlled bioprinting process, while maintaining the same thermal history on both biological samples. Figure 8 reports good cell viability after 4 h of culture, with a percentage of alive cells higher than 80%. This value is comparable to the one reported by the control. Therefore, the bioprinting process allowed us to bioprint more complex and bigger constructs without impairing cell viability. Moreover, the results obtained suggested that this strategy is also compatible with the bioprinting of organoids.

## 4. Conclusions

In conclusion, Matrigel^®^ is the most used material in cancer research, thanks to its biologically active nature. However, its use in large-scale constructs is still limited due to its complex rheological behavior and low mechanical properties. Indeed, it is not possible to extrude Matrigel^®^ with a standard pneumatic-driven dispensing system as it is abruptly ejected out of the syringe as soon as pressure is applied. In particular, this uncontrollable behavior prevents its use in bioprinting as pure bioink. However, Matrigel^®^ possesses peculiar biological characteristics, which presents biologists with the requirement of novel systems to produce free-standing, large-scale constructs with pure Matrigel^®^. Therefore, we modified a commercially available 3D FDM printer, in order to substitute the original extruder with a volumetric dispensing system for bioink extrusion. It was demonstrated to be an effective method to bioprint pure Matrigel^®^ constructs and to maintain a certain degree of shape fidelity, even when printing on multiple layers. The modifications to the 3D printer allowed us to accurately control the flow rate of the bioink during the printing process, maintaining a good level of cell viability.

## Figures and Tables

**Table 1:** Gcode used at the start and at the end of the print. Some gcode lines were modified or removed.

Starting Gcode	Ending Gcode
G21; metric values	
G90; absolute positioning	
M82; set extruder to absolute mode	G91; relative positioning
M107; start with the fan off	G1 Z25.0; move Z up
G28 X0 Y0; move X/Y to min endstops	G1 X0 Y0;
G28 Z0; move Z to min endstops	M84; steppers off
G1 Z25.0 F9000; move the extruder up	G90; absolute positioning
* M302 S0; (always allow extrusion)	
* M92 E150;	
* G1 F9000;	

* Asterisks indicate modifications to the original gcode file.

**Table 2:** Indexes used to evaluate printability for multi-layered structures.

3D Printed Structure	Shape Fidelity [%]	Layer Height [mm]	Structural Integrity [%]	Mean Fiber Diameter [mm]
Square	33.83 ± 8.98	1.41 ± 0.43	58.69	2.71 ± 0.41
Circle	27.58 ± 9.08	1.45 ± 0.43	60.34	3.40 ± 0.74

## Data Availability

Data sharing not applicable.
